# Efforts to Secure Nicotine and Cannabis Product Placements in Popular Media by Ploom, Pax, and Juul: An Analysis of Tobacco Industry Documents

**DOI:** 10.1093/ntr/ntaf180

**Published:** 2025-09-03

**Authors:** Tanner Wakefield, Stella Bialous, Pamela Ling, Dorie E Apollonio

**Affiliations:** School of Medicine, University of California, San Francisco, CA; School of Nursing, University of California, San Francisco, CA; School of Medicine, University of California, San Francisco, CA; School of Pharmacy, University of California, San Francisco, CA; Global Health Centre, Graduate Institute of International and Development Studies, Geneva, Switzerland

## Abstract

**Introduction:**

Tobacco companies advertised cigarettes in popular media to increase social acceptability. Most research on e-cigarette product placement has been content analyses of music videos. We explored the motivations, activities, and results of product placement activities of the e-cigarette company Ploom and its successors, Pax Labs and Juul Labs, in music videos as well as film and television.

**Methods:**

Case study using internal industry documents released in litigation and housed at the UCSF Truth Tobacco Industry Documents Library. Relevant documents were cataloged, summarized, and placed in chronological order.

**Results:**

We identified 451 relevant documents. Ploom sought product placements to expand its audience and enhance credibility. After Ploom split into Pax Labs and Juul Labs, they pursued free product placements by establishing relationships with prop masters in the entertainment industry and loaning devices to media productions using a placement agency. Later, Pax devices secured placements in music videos, film, and television; Juul appeared to secure only one placement.

**Conclusions:**

Ploom and its successor companies secured free product placements in music videos, films, and television. Pax devices associated with cannabis were placed more frequently than Juul. Given that tobacco advertising causes youth initiation, stronger regulation of product placement practices may be warranted.

**Implications:**

Ploom, Pax Labs, and Juul Labs used entertainment industry firms and outreach to prop masters to obtain numerous free product placements as consumers in general increasingly ignore or avoid traditional advertising. Product placements, which can improve brand recall, are better liked than commercials, and contribute to youth smoking uptake, appeared in popular and culturally relevant movies, shows, and music videos. E-cigarette industry product placement practices reflected those successfully used by major tobacco companies in the past.

## Introduction

Product placement is a form of advertising that can normalize tobacco products and promote consumption. In the 1980s, tobacco companies pursued product placement in films to promote the social acceptability of tobacco use.^1^ Tobacco product depictions in film and television increase youth smoking initiation^2-4^ and youth are more likely to recall tobacco product placements than online advertisements.^5^ Product placements are received more positively than commercials,^6^ improve brand recognition, improve attitudes toward a brand,^6^^,^^7^ and may increase the likelihood of purchase.^6^ A 2022 study found that branded cigarette product placements increased demand and that eliminating such depictions would reduce cigarette purchases by 2%.^8^ Product placements make it possible for companies to maintain access to viewers who use technological workarounds (e.g. ad blockers) or alternative access models (e.g. ad-free versions) to avoid traditional advertisements.^9^

The 1998 Master Settlement Agreement (MSA)^10^ prohibited tobacco companies that were party to the settlement from obtaining paid product placement in films and television.^11^ However, the MSA does not affect companies outside of the agreement, including e-cigarette companies.^10^^,^^12^ E-cigarettes have had an increasing share of the tobacco market since their introduction in 2007.^13^ E-cigarettes are addictive, expose users to harmful substances, including heavy metals, chemicals, and volatile organic compounds,^14^ and are associated with adverse health outcomes.^15-18^ In 2024, e-cigarettes were the most popular nicotine product among people^14^ under age 18 in the United States,^13^ with 10% of high school students using them in 2023,^19^ a reduction from the high of 27.5% in 2019.^13^

Content analyses of e-cigarette product placements in media have found that tobacco and e-cigarettes commonly appear in hip-hop music videos,^20^ generating over a billion exposures to e-cigarettes.^21^^,^^22^ Exposure to e-cigarette product placements in music videos is associated with an increased likelihood of considering use^23^ and past 30-day consumption, with odds of use increasing with the amount of product placement content seen, and higher among those younger than 21 years.^24^

Past academic research assessing e-cigarette product placements has focused primarily on e-cigarette imagery in Netflix content.^25-27^ We were unable to identify previous studies examining why or how e-cigarette companies pursue product placements in media, or how placements were valued as an advertising method. A 2023 study found few or no restrictions on depicting tobacco and cannabis products related to film and television incentives.^28^ However, this study did not address industry motivations for e-cigarette product placements.

**Figure 1:**
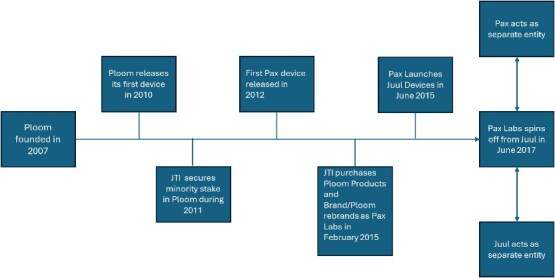
Timeline of Ploom’s evolution into Pax Labs and Juul Labs.^29^^,^^38^^,^^81^^,^^118^  ^*^Pax Labs and Juul Labs operated as separate entities but legally remained one company.^38^

In this study, we focus on Ploom,^29^ which initially released a heated tobacco product that used pods.^30^^,^^31^ In 2012, Ploom released another product line, Pax, a device marketed as a way to vaporize loose-leaf tobacco^31-33^ that quickly became associated with cannabis use^34^^,^^35^ (as of 2024, Pax marketed its devices as cannabis vaporizers^36^). Ploom became Pax Labs in 2015^32^^,^^37^ and began selling Juul devices (e-cigarettes containing nicotine) in 2015. Juul Labs formed as a spin-off in 2017^38^ (Figure 1). Pax Labs and Juul Labs sold their eponymous products while appearing to be separate companies, despite legally remaining one company.^38^

We investigated the product placement practices of Ploom and its successor companies, Pax Labs and Juul Labs,^29^^,^^38^ to determine the motivations, strategies, and outcomes related to product placement in popular media.

## Methods

This case study relied on previously secret internal Juul documents archived at the UCSF Truth Tobacco Industry Documents Library, a repository of documents from major tobacco and e-cigarette companies that have been made publicly available via litigation.^39^^,^^40^ We searched the library between March 2024 and January 2025. Initial keyword searches included “product placement,” “marketing placement,” and “paid placement.” We used snowball searches, adding additional terms and names discovered during our initial investigation.

One author (TW) with over 5 years of industry document research experience conducted an initial review of all the documents identified through keyword searches, excluding documents that did not contain relevant information (ie, documents that did not reference product placement decisions). Key concepts in these documents were identified inductively by all the authors during weekly meetings in 2024–2025. Preliminary coding was verified by three additional authors (SB, PL, and DA), each of whom had at least 20 years of experience in industry document research. In the case of disagreements, the entire team discussed the documents in question until reaching consensus. The documents determined to be relevant by the team were organized by keyword in a shared Excel spreadsheet that contained identifying information and content summaries of each document and then subsequently organized chronologically to understand the course of product placement activities over time. We used dates provided by the Truth Tobacco Industry Documents Library as estimated dates when a date was not embedded in a document. We based this analysis on approximately 451 documents dated 2007–2019, 74 of which are cited in this paper; these 74 documents were selected for inclusion in the manuscript because they offered the most complete information relevant to product placement decisions and did not duplicate other relevant documents.

## Results

### Product Placement Activities Begin

A Ploom marketing document estimated to be from 2007 indicated product placement marketing was part of the effort to have “… Ploom woven into popular culture – movies, tv, etc organically” by forming relationships with people connected to celebrities to encourage them to use the product.^41^ Ploom marketers hoped that providing free products to celebrities, providing customized versions, and supporting their charitable causes would eliminate the need to pay for product placement and earn free media over time.^41^

By February 2009, Ploom had expanded this strategy with a three-phase plan for marketing.^42^ The first phase focused on product “seeding,” or gifting, to influential people, the second on participating in events such as tasting rooms, and the third on product placements.^42^ Ploom marketers hoped to gain placements by providing products to directors, writers, and others in the film industry.^42^ In a November 2009 marketing document, Ploom employee Lily Ngo suggested gifting products to influencers in film, music, fashion, and other creative industries.^43^ Ngo also proposed providing samples at movie premieres, concerts, and art openings to reach arts, music, and entertainment influencers and celebrities.^43^ She considered musicians the company’s main target, believing music crossed socioeconomic barriers, reflected product/company traits, and that musicians regularly used different substances.^43^ Ngo felt that the film and television stars on her target list received significant media coverage and could be expected to try a “better” product than smoking without compensation,^43^ and expected that the people involved in fashion believed smoking helped manage weight and mitigate hunger.^43^

### Establishment of Voluntary Marketing Restrictions

By 2012, Ploom had established internal marketing standards that could be applied to product placements.^44^ A copy of the Ploom Marketing Code, estimated to be from 2012, does not reveal motivations, but shows that the company’s standards required that advertising could not depict persons under the age of 25 using Ploom products^44^ and should not be attractive to youth.^44^ Ploom products could not be marketed as increasing “sexual success,” could not infer that a majority of people smoked, or be tied to career success, popularity, or athletic achievement.^44^

### Ploom Hires a Product Placement Agency

In an unsigned copy of a 2013 contract, Lucey Stepp was tasked with helping Ploom network with media figures and land product placements.^45^ Stepp Up Marketing subsequently became Lucey Stepp (the last names of the agency’s major partners, Brian Lucey and Ariel Stepp^46^). From 2012 to 2014, Ploom paid Lucey Stepp $216 854^47-49^ for product placement–related services ($292 110 in 2024 dollars) (Table 1).

**Table 1:** Product Placement Spending

		2012	2013	2014	2015	2016	2017	2018
Film ($)	Original							
	2024 dollars							
Television ($)	Original			7500				
	2024 dollars			9962				
Music videos ($)	Original			20 500				
	2024 dollars			27 671				
Ploom/Pax device/company agency costs ($)	Original	3450	182 250	37 154	21 500	30 000	30 000	5000
	2024 dollars	4725	246 004	49 350	28 523	39 305	38 485	6261
Juul device/company agency costs ($)	Original	None	None	None	14 000	27 000	16 500	None
	2024 dollars	None	None	None	18 574	35 374	21 167	None
Total known spending ($)	Original	3450	182 250	65 154	35 500	57 000	46 500	5000
	2024 dollars	4725	246 004	86 983	47 097	74 679	59 652	6261

In July 2012, Lucey Stepp coordinated discussions on product placement pricing and appearance details for the season six finale of *Californication*, a then-popular HBO show that averaged 2.5–3 million viewers per episode.^50^ They planned to pay $10 000–$15 000 for a verbal mention and a product depiction; *Californication* also offered inclusion in related radio promotions and a live event.^50^ Ploom did not secure placement in the series finale but appeared in an episode that aired midseason during May 2014.^51^

Ploom and Lucey Stepp pursued music video appearances and collaborations with musicians. On December 13, 2013, Lucey Stepp secured a contract for a Pax product placement in a music video for Alexander Spit.^52^ After music group Weekend Money included two Pax devices in a 2014 music video,^51^^,^^53^ Ploom and Lucey Stepp collaborated with the artists to sponsor a listening party promoting their album.^54^ Ploom invited guests from magazines, music websites, and other media outlets to attend and planned to gift each a Pax device.^55^

Lucey Stepp also coordinated placements in films. In March 2014, Lucey Stepp helped arrange a product placement appearance within the movie *Entourage*, a spin-off of a popular HBO television program of the same name (Table 2).^56^ In April 2014, Lucey Stepp coordinated a product placement contract between Ploom and Fiction Pictures, resulting in a Pax appearance in the independent short film, *Liars Chair*,^57^ released publicly in 2019 (Table 2).^58^

**Table 2:** Pax and Juul Product Placements by Medium

	Music videos		Film		Television	
	Pax	Juul	Pax	Juul	Pax	Juul
2013	Aspektz—“Avion”^51^	None		None	*High Maintenance* // “Qasim” (HBO)^51^	
2014	Alexander Spit—“Millions”^45^^,^^51^ Weekend Money—“Clockworkin’”^45^^,^^51^ Mayer Hawthorne featuring Kendrik Lamar—“Crime”^51^^,^^62^^,^^119^ (316 000 impressions)^*^^51^^,^^90^ The Frail—“Automatic”^51^^,^^62^^,^^119^ Tuxedo—“Do It”^51^ Jhene Aiko feat. Childish Gambino—“Bed Peace”^51^ Tew Unon—“Stay Blunted”^51^ El-P—“Rhythm Roulette”^51^ White Arrows—“We Can’t Ever Die”^51^^,^^120^ Radiator Hospital—“Bedtime Story”^51^	None	*Creative Control* ^45^ *The Liars Chair* ^121^ *Clapping for the Wrong Reasons* (short film)^51^	None	*Broad City* ^45^ ^,^ ^119^ Lucas Brothers New Show^45^ *Looking* ^87^ (one episode—240 000 impressions)^*^^51^^,^^90^ *Person of Interest* ^87^ *Arsenio Hall Show* ^51^ *Californication* ^51^ *Getting Doug with High* (web show)^51^ *Eminem’s Shady Films Presents: Road to Total Slaughter,* Ep. 1^51^	None
2015	Childish Gambino—*Clapping for the Wrong Reasons*^120^ (14 500 impressions)^*^^90^	None	*Entourage* ^89^ ^,^^122^ (3.2 million impressions)^*^^90^ *Dope* ^89^ ^,^^122^ (610 000 impressions)^*^^90^	NONE	*High Maintenance* ^120^ (one episode—1 million impressions)^*^^90^ *Person of Interest* ^122^ (8 million impressions)^*^^90^ *Younger* ^89^ (one episode—1.16 million impressions)^*^^90^ *Weed 3* ^89^ *Broad City* (Pax and Juul)^89^ (five episodes—3.9 million impressions)^*^^90^ *Grace and Frankie* ^89^ *Benders* ^122^ *Getting Doug with High* ^74^ *Billions*	None
2016					*Broad City* ^123^ *Weediquette* ^123^ *High Maintenance* ^123^ *Strangers* ^123^ (Refinery 29 online series) *Billions* ^124^	
2017					*Girls* (HBO)^105^	*Girls* ^**^ ^125^

^*^Impressions as of August 26, 2015.

^**^First Juul placement.

### Paid Placements in 2013–2014

While Ploom sought no-cost product placements and continued to claim publicly that it did not pay for placements, a marketing document estimated to be from June 2014 set a budget of $325 000 ($431 687 in 2024 dollars) for placements in a bid to increase audience exposure and the brand’s standing.^59^ It included plans for appearance in music videos, including gifts to production staff,^49^^,^^60^ and movies.^49^ Ploom secured product placements in 11 music videos, 8 shows, and 3 films by the end of 2014 (see Table 2). Product placements in 2013 and 2014 generated at least an additional 44 spots in free media, eg, news or cultural articles regarding the media with Ploom product placements.^51^

Ploom continued to pay for product placements in 2014.^61^ Ploom’s field marketing manager,^62^ Kate Morgan,^63^ oversaw product placement, influencer engagement, and public relations to advance brand reach and viewership.^61^ In a 2014 uncredited marketing recap presentation, Ploom reported that product placement, including gifts to celebrities, increased public visibility, generated trust, positioned the product as aspirational, and built media interest.^64^ Heading into 2015, Ploom considered growing its brand presence as a “key initiative” that it would pursue partly via product placement.^65^

In September 2014, Ploom contracted Los Angeles–based agency Eclipse Worldwide to provide “product placement services”^66^ (after ending its relationship with Lucey Stepp for unstated reasons in July 2014^67^). Still operating as of 2024,^68^ Eclipse Worldwide specialized in product placements, loaning and tracking devices to media projects to secure appearances and monitor outcomes.^69^ Ploom also tasked the agency with capturing and providing it media content featuring Pax placements.^69^ Eclipse Worldwide helped organize and host parties to promote Pax products in 2014, which also promoted Juul products in 2015, to prop masters in the entertainment industry.^70-73^ Ploom paid Eclipse Worldwide a monthly retainer of $1500 ($1922 in 2024 dollars) from September to December 2014. (Table 1).^73^ By December 2014, Ploom had lent Pax to 10 episodic shows, 2 pilots, and 3 movie productions.^74^ Starting in January 2015, Ploom increased the Eclipse Worldwide monthly retainer to $2500 ($3317 in 2024 dollars).^75^ Eclipse Worldwide received at least $150 000 ($189 611 in 2024 dollars) between 2014 and 2018 for product placement services provided to Ploom, Pax, and Juul (Table 1).

### Ploom Outreach to Prop Masters

Ploom’s outreach to prop masters in the film and television industry also involved hosting parties to network and develop product placement opportunities. In October 2014, to promote Pax, Ploom held a party for 75–150 prop masters in Los Angeles^76^ representing shows such as *The Big Bang Theory*, *Keeping Up with the Kardashians*, *Glee*, and *Arrested Development*.^77^ While Ploom did not explicitly state the agency involved, Eclipse Worldwide received $1700 for “product placement and intro party expenses” in October 2014.^73^ In December 2014, Ploom held a second prop-master party with an “LA product placement agency”^78^ that provided access to 52 prop masters.^79^

### Ploom Becomes Pax After Selling the Rights to Its Name and Ploom Products

In February 2015, Japan Tobacco International, which had owned a minority position in Ploom since 2011,^80^ bought the rights to Ploom-branded products, which resulted in Ploom becoming Pax Labs (see Figure 1).^29^^,^^37^^,^^81^

#### PAX Labs

Pax Labs continued to pay Eclipse Worldwide $2500 ($3317 in 2024 dollars) per month until June 2015.^75^ When Pax Labs began product placement efforts for Juul, it switched to two separate monthly retainer payments: $1250 for Pax and $1250 for Juul ($1658 each in 2024 dollars).^72^^,^^75^ Pax Labs increased its product placement retainers with Eclipse Worldwide to $5000 ($6551 in 2024 dollars) total ($2500 each) ($3275 in 2024 dollars) by February 2016.^82^^,^^83^

### Outreach to Retailers to Expand Product Placement

Ploom’s relationships with retailers also helped secure product placements. A New York City–based retailer informed Ploom on January 6, 2015, that it had recommended Pax devices to CBS producers seeking e-cigarettes to include in an episode of *Criminal Minds*.^69^ Juul recognized that the retailer’s New York City stores, the Blue Nile and The Smoking Shop, were visited by television producers, rappers, and musicians.^84^

### 2015 Product Placement Strategy

In a February 2015 E-mail, Field Marketing Manager Kate Morgan^85^ indicated that Pax devices had secured placements in multiple projects because “… they are all relationship based and/or because people LOVE our product (Lucky for us people really, really, really love PAX…),” and the company made an effort to maintain these relationships.^74^ Loaning devices to projects increased the odds they were included in media but did not ensure it. Morgan stated in an April 14, 2015, E-mail that “… we aren’t guaranteed to be placed on these shows since we don’t pay to play … .”^86^ In April 2015, Pax Labs had 23 Pax devices on loan to episodic projects, including shows on major television networks.^87^ By July 2015, after Pax Labs released Juul devices,^88^ the company distributed Pax and Juul devices to an additional 33 film and episodic projects for potential product placement.^89^ From these loans, Pax devices secured placements in 11 shows and 2 films in 2015 and 5 shows in 2016; Juul devices secured no placements (see Table 2). In August 2015, Pax Labs determined that their product placements in 10 shows, movies, and music videos had generated approximately 21 340 000 viewer impressions.^90^ In a September 2017 E-mail, Morgan, who had become Pax’s head of global marketing,^91^ stated that she usually passed on paid placements because she could generally get them for free.^92^ Mark Shin, a senior international marketing manager at the company, agreed, stating that Pax did not pay for placements except for instances where it provided content use access and “massive exposure.”^92^

### Pax: Prop Party Distribution in 2015

Prop-master parties continued in 2015. Pax Labs planned events in Los Angeles and New York in May 2015 to promote Pax and the new Juul devices^93^ to be launched in June 2015.^88^ Morgan oversaw the prop-master parties,^94^ and planned to hold the events twice a year in both cities as rolling events.^74^^,^^95^ Morgan stated in a January 27, 2015, E-mail that the New York prop-master party was being organized “… so they feel inclined to use PAX & JUUL in product placement … .”^93^ The Los Angeles and New York City prop-master parties ultimately occurred in June 2015 at the Henley in New York City and Eclipse Worldwide in Los Angeles,^70^^,^^71^ with the former “hosted” by Juul.^96^ Pax Labs planned to gift Pax devices to prop masters attending the parties and provided Juul starter kits to 30–40 prop-master attendees at the Henley.^96^^,^^97^ In total, 87 people attended the June 2015 parties.^98^ Pax spent at least $20 000 ($26 534 in 2024 dollars) for two prop-master parties held in New York and Los Angeles in 2015 (Table 1).

### Pax: Networking for Product Placements

Pax Labs also worked with media producers to secure product placements. In August 2015, Morgan secured specialty customer service for the executive producer of the movie *Entourage*, stating that “He is a big reason we get placed in some films/tv shows.”^99^ The producer had experienced “weak hits” from his Pax 2 vaporizer.^99^ He requested products to use, and because he had helped secure previous placements in other unspecified projects, Morgan provided them.^99^ Pax considered sending a device to a magazine editor’s partner who worked at Def Jam, since the editor suggested they could be used for product placement.^100^ In October 2015, Morgan entered dialog with a representative for Lorelei Shellist, a model, style expert, filmmaker, and author, who requested Pax or Juul devices for placement in her employer’s upcoming fashion movie.^101^ Morgan ultimately connected the representative with Pax’s product placement agency to obtain loans.^101^ These efforts contributed to Pax appearing in one music video, two movies, and nine shows during 2015 (Table 2).

### 2017: Juul Labs Spin-off

Pax Labs spun off Juul Labs as a separate entity in June 2017 to oversee Juul device sales and marketing (Figure 1).^38^ While Juul Labs appeared to be a separate company, it remained the same company as Pax Labs,^29^^,^^38^ with its own internal structure^102^^,^^103^ and outwardly separate corporate branding.^38^

### Decreasing Product Placements by 2017

Product placements dropped significantly for Pax Labs in 2016 and 2017 compared to 2014 and 2015 levels (see Table 2). When discussing pitches in a March 2016 E-mail, Pax Labs’ Sarah Richardson noted that product placement had become the least significant marketing avenue for the company because of its low effectiveness at converting people into customers.^104^ Pax products still appeared in five shows in 2016 and two shows in 2017, one of which also included Juul (Table 2). The joint placement of Pax and Juul in a 2017 episode of the HBO show *Girls* caused Kelly Evans of the Pax-affiliated PR firm Havas Formula to state in a March 23, 2017, E-mail that “Anyone watching this would think this is a paid placement. And Pax/Juul fans would instantly spot this, and others into smoking would jump on Google to figure out what they’re smoking.”^105^ The episode of *Girls* marked the first major media placement for a Juul device.^105^

### End of Product Placements

Juul made its last retainer payment to Eclipse Worldwide for product placement services in June 2017.^83^ According to a September 21, 2017, E-mail from Joanna Lenn of Eclipse Worldwide, the agency had decided it “… would not represent JUUL due to the sensitive issues with age, and the ability for productions to confuse JUUL and (Pax) ... .”^106^ Further, Juul may have stopped using product placement services because of limited success with placements for advertising; Morgan stated in an October 2017 E-mail that Pax was easy to place on television shows but that “... no one wants to place Juul … .”^107^ Pax Labs continued to pay a $2500 monthly retainer to Eclipse Worldwide until at least February 2018.^82^^,^^108^

Despite not managing product placement efforts, Eclipse Worldwide forwarded a product placement opportunity to Juul for the show *Power* on Starz, which the company pursued.^106^ However, Morgan later claimed in a September 26, 2018, document that Juul’s product placement agency had dropped the company because it avoided paying for placements.^109^ Juul still received device requests from at least two prop masters in a document estimated to be from October 2018.^110^^,^^111^

## Discussion

Ploom, the company that later became Pax and Juul, weighed product placements as a marketing strategy as early as 2007 and began securing significant placements by 2013. After succeeding Ploom in 2015, Pax Labs pursued product placements for Pax and Juul devices by hosting events, disseminating products to prop masters, and loaning devices to media projects with the assistance of an agency. After Juul Labs spun off from Pax Labs, it struggled to secure product placements and ceased efforts in 2017 due to an increasingly hostile public and regulatory climate.^112^ Following increasing public concern that Juul was marketing to youth with fruit and mint flavors, as well as product designs that made the devices easy to hide, the Food and Drug Administration (FDA) issued a marketing denial order in 2022 that would have removed Juul products from the US market (following a court challenge, the FDA agreed to a continuing review in 2024).^113-115^ In contrast, Pax Labs ended active placement efforts in 2018, concluding that the practice had become an ineffective marketing strategy. Given that Pax products became associated with cannabis use rather than nicotine, it is possible that attempting to secure placements in popular media was more challenging, given that legalization of recreational cannabis use at the state level had just begun in the United States at the time.

In the 2010s, Ploom, Pax Labs, and Juul Labs primarily pursued free product placements, a standard practice in the entertainment industry, as more consumers ignored or skipped advertisements.^116^ Most placements in the US film and television industry are not paid and rely on loaned products to reduce costs.^116^ Ploom and its successors made few paid placements relative to free placements, although the company claimed publicly that they did not pay for placement at all. However, the activities and funding needed to obtain these unpaid depictions were substantial. The past inability of outside observers to identify paid placements for Ploom, Pax, and Juul is mirrored in 2020s concerns about the extensive exposure to Zyn nicotine pouches, which are promoted by TikTok influencers who do not disclose whether these are paid placements.^117^

Ploom, Pax Labs, and Juul Labs pursued product placements in the same ways tobacco companies did historically. According to a 2002 case study, major tobacco companies pursued free product placements in the 1980s, believing that they were more impactful than traditional advertising and effective with youth, that being associated with movies left positive impressions on viewers, and that they could function as a means to maintain the social acceptance of smoking.^1^ Ploom viewed product placements as a way to integrate itself into popular culture,^41^ as well as expand visibility and establish legitimacy.^59^ The product placement tactics used by Ploom, Pax, and Juul Labs were similar to past tobacco company strategies. RJ Reynolds had gifted products to celebrities and influential industry members to increase the odds of appearing in films^1^; Philip Morris, Brown and Williamson, and American Tobacco relied on agencies to pursue and coordinate product placements in film and television.^1^

A strength of this paper is its use of internal documents to reveal the motivations and activities of Ploom and its successors regarding product placement. Although previous studies have explored product placement strategies for combustible cigarettes, and e-cigarettes in music videos, this study is the first to examine e-cigarette industry placement practices in the 2010s. However, the archive is incomplete; our investigation of e-cigarette product placements in music videos, films, and television is limited to placements secured by Ploom, Pax Labs, and Juul Labs. Future research that explores e-cigarette product placements overall, particularly in film and episodic content such as streaming and television shows, would help determine to what extent these results are generalizable.

## Conclusions

Ploom and its successors secured free product placements in music videos, films, and episodic content by loaning devices and networking with prop masters and industry figures. These findings suggest that stronger regulation is needed to prevent e-cigarette product placement. Tracking free and paid product placements would aid in understanding reach and impact on the use of e-cigarettes. Legislation that disincentivizes e-cigarette product placements, such as restrictions on receiving funds from state film incentive programs, could also discourage placements.^28^

## Data Availability

The data supporting the conclusions of this article are publicly available in the Truth Tobacco Industry Documents library.
